# Development of an Androgen Receptor Inhibitor Targeting the N-Terminal Domain of Androgen Receptor for Treatment of Castration Resistant Prostate Cancer

**DOI:** 10.3390/cancers13143488

**Published:** 2021-07-12

**Authors:** Fuqiang Ban, Eric Leblanc, Ayse Derya Cavga, Chia-Chi Flora Huang, Mark R. Flory, Fan Zhang, Matthew E. K. Chang, Hélène Morin, Nada Lallous, Kriti Singh, Martin E. Gleave, Hisham Mohammed, Paul S. Rennie, Nathan A. Lack, Artem Cherkasov

**Affiliations:** 1 Vancouver Prostate Centre, University of British Columbia, 2660 Oak Street, Vancouver, BC V6H 3Z6, Canada; fban@prostatecentre.com (F.B.); eleblanc@prostatecentre.com (E.L.); fhuang@prostatecentre.com (C.-C.F.H.); fzhang@prostatecentre.com (F.Z.); hmorin@prostatecentre.com (H.M.); nlallous@prostatecentre.com (N.L.); ksingh@prostatecentre.com (K.S.); mgleave@prostatecentre.com (M.E.G.); prennie@prostatecentre.com (P.S.R.); nlack@prostatecentre.com (N.A.L.); 2 School of Medicine, Koç University, Rumelifeneri Yolu, 34450 Istanbul, Turkey; acavga13@ku.edu.tr; 3 Knight Cancer Institute, Oregon Health & Science University, 3181 S.W. Sam Jackson Park Rd., Portland, OR 97239-3098, USA; flory@ohsu.edu (M.R.F.); changma@ohsu.edu (M.E.K.C.); mohammeh@ohsu.edu (H.M.); 4 Koç University Research Centre for Translational Medicine (KUTTAM), Koç University, Rumelifeneri Yolu, 34450 Istanbul, Turkey

**Keywords:** castration resistant prostate cancer, androgen receptor inhibitor, N-terminal domain, small molecule inhibitor, computer-aided drug design, AR splice variants

## Abstract

**Simple Summary:**

In almost all patients, the proliferation and growth of prostate cancer is driven by the androgen receptor. Given this critical role, inhibiting the androgen receptor is the standard of care following surgery or radiotherapy. However, the effectiveness of current anti-androgen therapeutics is almost always temporary, with the cancer eventually developing drug resistance. There are no curative treatment options for resistant prostate cancer and these patients have an average life-expectancy of only 20 months. Currently, all clinical inhibitors of the androgen receptor act by direct binding to the hormone binding site. These drugs are therefore vulnerable to mutations which frequently arise at this region or to the emergence of truncated forms of the androgen receptor that are constitutively active without any need of hormone binding. In this study, we aim to develop small molecules inhibitors that attack the molecular functions of the androgen receptor at the N-terminal domain, distinct from steroid recruitment to evade resistance to conventional treatments.

**Abstract:**

Prostate cancer patients undergoing androgen deprivation therapy almost invariably develop castration-resistant prostate cancer. Resistance can occur when mutations in the androgen receptor (AR) render anti-androgen drugs ineffective or through the expression of constitutively active splice variants lacking the androgen binding domain entirely (e.g., ARV7). In this study, we are reporting the discovery of a novel AR-NTD covalent inhibitor 1-chloro-3-[(5-([(2S)-3-chloro-2-hydroxypropyl]amino)naphthalen-1-yl)amino]propan-2-ol (VPC-220010) targeting the AR-N-terminal Domain (AR-NTD). VPC-220010 inhibits AR-mediated transcription of full length and truncated variant ARV7, downregulates AR response genes, and selectively reduces the growth of both full-length AR- and truncated AR-dependent prostate cancer cell lines. We show that VPC-220010 disrupts interactions between AR and known coactivators and coregulatory proteins, such as CHD4, FOXA1, ZMIZ1, and several SWI/SNF complex proteins. Taken together, our data suggest that VPC-220010 is a promising small molecule that can be further optimized into effective AR-NTD inhibitor for the treatment of CRPC.

## 1. Introduction

Prostate cancer (PCa) is one of the most common forms of cancer in men, with over 1.3 million new cases per year worldwide [1]. Patients with recurrent or metastatic forms of the disease are commonly treated with therapies designed to inhibit the androgen receptor (AR) [2]. However, these are only temporarily effective, and the vast majority of patients will develop resistance. Once the disease has progressed to castration-resistant prostate cancer (CRPC) it is almost universally lethal. Yet, even in these resistant patients, the AR still remains the major driver of growth and proliferation [2]. How the AR remains active in either castrate conditions or in the presence of antiandrogens has been an active area of research and several common mechanisms of resistance have been identified, including: AR overexpression, somatic point mutations, intratumoral androgen production, or constitutively active AR variants (ARVs) [2,3,4]. Lacking a ligand binding domain (LBD) these ARVs do not require androgen to be activated [5] and are therefore resistant to current therapeutics [6].

Most, if not all, of the AR transcriptional activity is mediated via the N-terminal domain (NTD) [7,8]. Given this critical function, targeting the NTD offers a promising strategy to overcome common mechanisms of resistance in CRPC patients, including ARV and somatic point mutations. However, as the NTD is highly disordered, this site was previously thought to be “undruggable”. Work by Sadar and Andersen demonstrated that despite the disorder, small molecule NTD inhibitors can be developed against this challenging target [9,10,11]. From these compounds, EPI-506 progressed into clinical trials for metastatic CRPC but was terminated due to poor pharmacokinetics that necessitated a very high pill burden [12]. However, even with low levels of circulating EPI-506, there was partial pharmacodynamic efficacy with a 4–29% decrease in PSA, an AR regulated gene. These studies demonstrated that targeting the NTD, while being challenging, offers a potential mechanism to inhibit AR signaling in CRPC patients.

Here, we have developed novel AR NTD inhibitors that were extensively characterized in biological assays. We show that these compounds effectively inhibit both full length AR and its constitutive variant ARV7 by disrupting critical coactivator interactions. Of these, VPC-220010 demonstrated superior inhibitor activity in AR transcription and AR-dependent growth of cancer cells compared to EPI-001. While still needing further hit-to-lead optimization, the prototype AR NTD inhibitor VPC-220010 could provide avenue to overcome common CRPC resistance mechanisms by decreasing prostate cancer cell growth through an AR-directed mechanism of action.

## 2. Materials and Methods

*Medicinal Chemistry and synthesis of the VPC-220XXX series.* Detailed protocols for the synthesis of the VPC-220010 enantiomers can be found in Supplementary Materials.

*Cell culture:* LNCaP, PC3 and 22Rv1 human PCa cells were obtained from the American Type Culture Collection and authenticated by IDEXX Laboratories (Westbrook, ME, USA). Cells were maintained in RPMI-1640 media (Life Technologies, Carlsbad, CA, USA) and 5% FBS (Hyclone Thermo Fisher Scientific, Waltham, MA, USA). All cells were cultured at 37 °C and 5% CO_2_ and monitored weekly for mycoplasma contamination.

*AR fluorescent reporter assay:* Stably transfected eGFP-expressing LNCaP human prostate cancer cells (LN-ARR2PB-eGFP) containing an androgen-responsive probasin-derived promoter (ARR2PB) were assayed, as previously described [13].

*Luciferase assay:* For both AR and nuclear receptor assays, PC3 human PCa cells grown in RPMI 1640 media (Invitrogen) supplemented with 5% charcoal-stripped serum (CSS) were seeded in 96-well plates (5000 cells/well) and transfected with 50 ng of AR, estrogen receptor alpha (ER-alpha), glucocorticoid receptor (GR), or progesterone receptor (PR) plasmids, 50 ng of ARR_3_tk-luciferase (AR, GR and PR) or 3ERE-luc (ER) using 0.3 μL/well TransIT20/20 transfection reagent (TT20, Mirus). Following 48 h incubation, cells were then treated with compounds at various concentrations and 0.1 nm R1881 (in 100% ethanol) for 24 h for AR assays. GR, ER, and PR activation was stimulated with 1 nm dexamethasone, estradiol, or progesterone, respectively. For NTD-transactivation assays, LNCaP cells were co-transfected with 50 ng of both Gal4UAS-luciferase and AR (1-558)-Gal4DBD 24 h prior to treatment with forskolin (50 μM). Cell lysis was carried out with 60 μL of 1× passive lysis buffer/well (Promega Madison, WI, USA). 20 μL of cell lysate from each well were mixed with 50 μL of luciferase assay reagent (Promega Madison, WI, USA), and luminescence was recorded on a TECAN M200pro plate reader.

*Proliferation assay*: LNCaP, 22Rv1 and PC3 cells were plated at 3000 cells per well in RPMI1640 containing 5% CSS in a 96-well plate, treated with 0.1 nm R1881 (a synthetic androgen) and test compounds (0–25 μM) for 72 h. Cell proliferation was determined using the PrestoBlue^TM^ cell proliferation assay following incubation with the compound (0–100 μM) over 72 h. In brief, 30 μL of the PrestoBlue^TM^ reagent was added to cells in each well of the 96-well plate containing 200 μL of media and incubated for 60 min at 37 °C in 5% CO_2_. The production of formazan was measured at 490 nm. Fluorescence measurement was read on a TECAN F200.

*qPCR and RNA sequencing analysis:* LNCaP cells (6 × 10^6^) were seeded in a 10 cm plates and grown with RPMI 1640 media containing 5% CSS and antibiotics for 48 h. On the day of treatment, CSS media was refreshed and the LNCaP cells were treated with either EtOH + DMSO (EtOH Control), 10 nM DHT + DMSO (DHT Control), EtOH + VPC220010/ENZA, or DHT + VPC220010/ENZA for 6 **h**. The compounds were dissolved in 100% DMSO, using the IC_90_ concentration of VPC-220010 (6 µM) and Enzalutamide (1.5 µM). Total RNA was extracted from cells with Trizol and the Qiagen RNeasy RNA Extraction Kit (Qiagen Germantown, MD, USA). Approximately 8 × 10^6^ cells were collected per sample, with three biological replicates for each treatment. The concentrations and the qualities of the RNA samples were checked by Nanodrop. cDNA was prepared busing MMLV-Reverse Transcriptase (Thermo Fisher Scientific, Waltham, MA, USA). qRT-PCR was performed with LightCycler**^®^** 480 SYBR Green I Master (Roche, Basel, Switzerland). AR full length regulated genes TMPRSS2, FKBP5 and KLK3 in LNCaP cells, AR variant 2 regulated genes UBE2C, AKT1, CDC20 in PC3 cells (Supplementary Materials).

For RNAseq, the resulting total RNA was subjected to Poly-A selection, converted to cDNA and then sequenced with a BGISeq500. Reads were mapped to hg38 and quantified using Salmon version 1.1.0 [14], using—validateMappings—seqBias—gcBias parameters for high accuracy. Differential gene expression analysis for different treatments was then performed with DESeq2 [15] after filtering out low count reads. Pre-ranked Gene Set Enrichment Analysis [16] of MSigDB “hallmark” signatures were performed using the protein coding gene lists ranked by −log10 P value multiplied by the sign of log2-transformed fold-change [17]. The raw sequencing data can be found under GEO accession number GSE173331 and DESeq2 results are in  Supplementary Table S1.

*Rapid immunoprecipitation mass spectrometry of endogenous proteins (RIME):* LNCaP cells (4 × 10^7^/treatment) were cultured in RPMI-1640 medium supplemented with 5% CSS and 1% penicillin/streptomycin for 48 h. After refreshing the CSS culture medium, cells were treated with the same conditions as RNAseq samples for 6 h. After treatment cells were cross-linked by incubating in freshly prepared serum-free RPMI-1640 medium (no serum) containing 1% (*vol*/*vol*) formaldehyde for 8 min at room temperature on shaker. The reactions were then quenched with 0.1M glycine for 5 min. After two ice-cold PBS washes, cells were harvested by gentle scrapping in ice-cold PBS containing 100X protease inhibitor cocktail. The crosslinked AR protein complex was enriched with an AR antibody (Millipore # 06-680). From the resulting mass spectrometry data, the AR interactome was defined as those proteins that had >1 log2 fold-change in label-free quantification (LFQ) intensity in both DHT replicas compared to the ethanol control. Those proteins with a positive LFQ intensity in any IgG control samples were eliminated. A total of 111 proteins were identified to interact with AR in the DHT treated samples. Known AR interacting proteins were identified from previous published studies [18]. An interaction was determined to be disrupted by VPC-220010 or enzalutamide if there was a >1 log2 fold-change in LFQ from both replicas in DHT compared to compound treated sample. The resulting protein lists are detailed in Supplementary Table S2. The 67 proteins specifically disrupted by VPC-220010 were uploaded into the GSEA web server to calculate hypergeometric distribution probabilities of genes overlapping with Hallmark and Gene Ontology gene sets. The significance value threshold was set as an FDR q-value less than 0.05, which is the significance value corrected for test of multiple hypotheses.

*AR Phase separation:* The mEGFP-tagged AR plasmid was transfected into Charcoal Stripped Serum (CSS) starved LNCaP cells for 2 days using Lipofectamin 3000 (Invitrogen Life Technologies Carlsbad, CA, USA), following the user guide. Cells were stimulated with 1 nM dihydrotestosterone (DHT) for 2 h and then treated with 10 µM VPC-220010 or EPI-001 AR for an additional 2 h. Fluorescence staining was visualized with an Olympus FV3000RS confocal microscope (Olympus, Tokyo, Japan) using a 60× UPLAPO oil objective. The images were taken with the FV31S-SW software with the confocal pinhole set to “automatic” under the Z-stack model. The condensates were quantified by two independent investigators and presented as the percentage of cells containing the condensates to the cells expressing GFP-tagged proteins. The results shown are the average of three independent experiments.

## 3. Results

### 3.1. Determination of VPC-2055 as an AR-NTD Inhibitor

**Figure 1:**
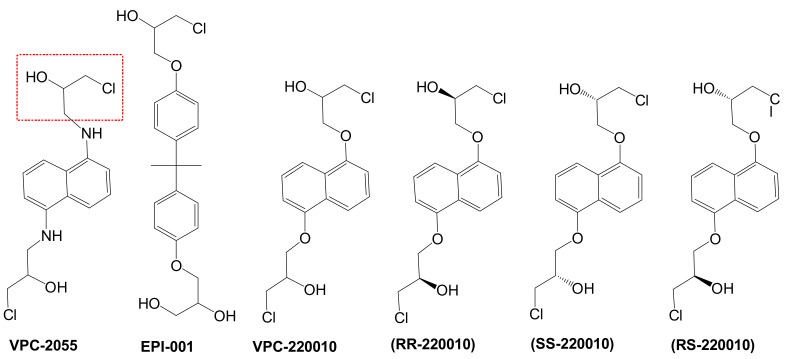
The chemical structures of VPC-2055, EPI-001, VPC-220010 and its enantiomers (RR-220010, SS-220010, RS-220010).

**Figure 2:**
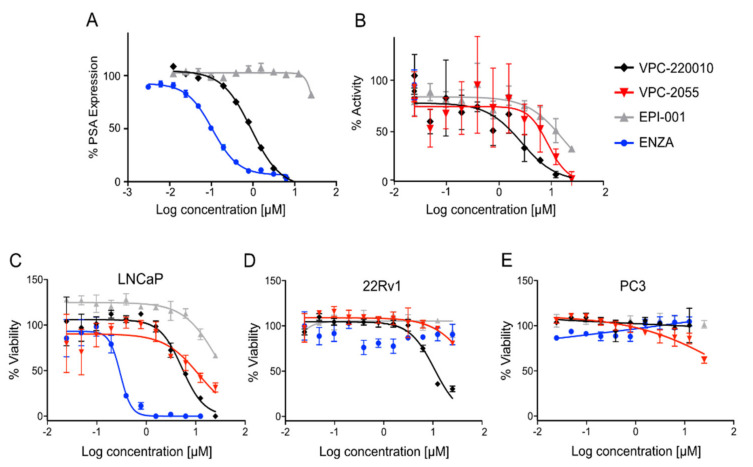
Effect of VPC-2055, EPI-001 and VPC-220010 on the transcriptional activity of (**A**) full-length AR in an eGFP reporter cell line; (**B**) ARV7 with a nanoluc reporter cell line; and the proliferation of (**C**) LNCaP (full length AR); (**D**) 22rv1 (full length AR and ARV7); and (**E**) PC-3 (AR null). Viability was calculated following 72 h stimulated by R1881 (DMSO vehicle: LNCaP), or with DMSO alone (22rv1, PC-3). All experiments were done as three biological replicas in triplicate (mean ± SD).

In previous work we demonstrated that in a panel of novel AR inhibitors many were active via an AR degradation mechanism [19]. However, one of these compounds, VPC-2055, was effective without any signs of AR degradation. Structural comparison between VPC-2055 and EPI-001 revealed that these two compounds share a chemically reactive moiety of 1-chloropropan-2-ol marked by a rectangular frame (Figure 1), which led us to suspect that VPC-2055 was inhibiting AR by binding to the AR-NTD. Using a Doxycycline-inducible PC3-ARV7 transcriptional activity assay [20] we confirmed that VPC-2055 was indeed active against this truncated variant (Figure 2B). In addition, we have found that VPC-2055 could effectively inhibit the proliferation of 22RV1, an ARV7-driven cell line (Figure 2C). Therefore, using VPC-2055 as a starting point to expand the naphthalene series, we synthesized and tested 110 novel compounds.

### 3.2. Identification of VPC-220010 as a Pan-Inhibitor of the AR

The newly synthesized compounds were screened initially for their ability to inhibit ARV7 transcriptional activity using a cell based ARV7 transcriptional assay. Of these, eight were found to be active under 10 uM (Table 1). Of all compounds tested, VPC-220010 was best at inhibiting ARV7 activity with an IC_50_ of 2.7 μM, which is approximately three and five times more potent than VPC-2055 (IC_50_ = 8.8 μM) and compound EPI-001 (IC_50_ = 17.1 μM), respectively (Figure 2B). Interestingly, while VPC-220010 is a racemic mixture, there was no difference in potency for the pure enantiomers RR-220010, SS-220010, and RS-220010 (Table 1). To rule out direct effects of the compounds on luciferase expression, we confirmed that the compounds were inactive in a Dox-inducible nanoluciferase PC3 cell line (no ARV7) [20].

Next, we tested the activity of VPC-220010 against full length AR using an AR-eGFP transcription assay in LNCaP cells [13,21,22,23]. VPC-220010 activity (IC_50_ = 0.7 uM) was comparable to VPC-2055 (IC_50_ = 1 uM) but 20 times better than EPI-001 (IC_50_ > 25 uM). To further validate VPC-220010 as an AR inhibitor, we evaluated its ability to reduce the production of endogenous prostate specific antigen (PSA) in PCa cell lines. PSA is an AR-regulated serine protease and is widely used as a biomarker for PCa. As expected, VPC-220010 reduced the secreted PSA levels in LNCaP cells with an IC_50_ of 0.91 μM. In contrast, EPI-001 had an IC_50_ value > 25 μM (Figure 2A).

**Figure 3:**
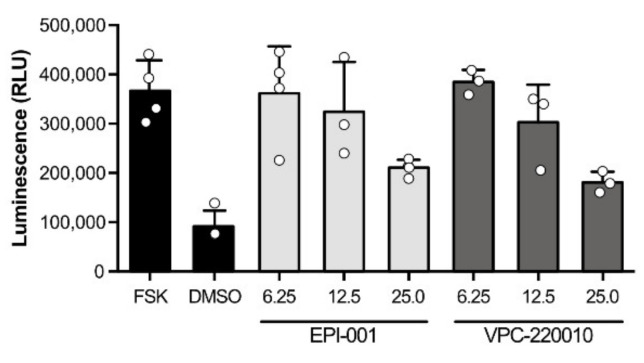
The AR-NTD activation by forskolin was inhibited by EPI-001 and VPC-220010 in a mammalian two hybrid assay. LNCaP cells were co-transfected with AR-NTD fused to a Gal4 DNA binding domain (Gal4DBD-AR_1-558_) and a reporter gene cloned downstream of the Gal4-binding site before treatment with forskolin (FSK) and varying concentrations of EPI-001 or VPC-220010.

To confirm the proposed mechanism of VPC-220010 we tested the effect of this compound on NTD-mediated transactivation. Using a mammalian two hybrid system, we co-transfected AR-NTD fused to a Gal4 DNA binding domain (Gal4DBD-AR_1-558_) with a reporter gene cloned downstream of the Gal4-binding site. When incubated with forskolin, this system induces transcription activity through phosphorylation of the AR-NTD [10]. We observed that the AR-NTD activity induced by forskolin stimulation was inhibited when treated with varying concentrations of both VPC-220010 and EPI-001 (Figure 3). Overall, these results demonstrate that VPC-220010 is acting through the AR-NTD.

We then evaluated the effect of VPC-220010 on the proliferation of androgen-dependent LNCaP and ARV7-dependent 22Rv1 cells. As expected, VPC-220010 caused a significant decrease in the viability of LNCaP cells, with an IC_50_ of 5.3 μM compared to VPC-2055 (11.8 μM) and EPI-001 (>25 μM) (Figure 2C). Further, VPC-220010 was also significantly more effective at reducing viability of 22Rv1 cells, with an IC_50_ of 10.8 μM, which was at least 5× more potent than VPC-2055 and EPI-001 (IC_50_ > 50 μM) (Figure 2D). We did not observe any significant effect on the viability of AR-negative PC3 cell line when incubated with VPC-220010 (Figure 2E). Overall, these results demonstrate that VPC-220010 can selectively inhibit full length and truncated AR and reduce AR-mediated PCa cell viability.

### 3.3. Specificity of VPC-220010 for the Androgen Receptor

**Figure 4:**
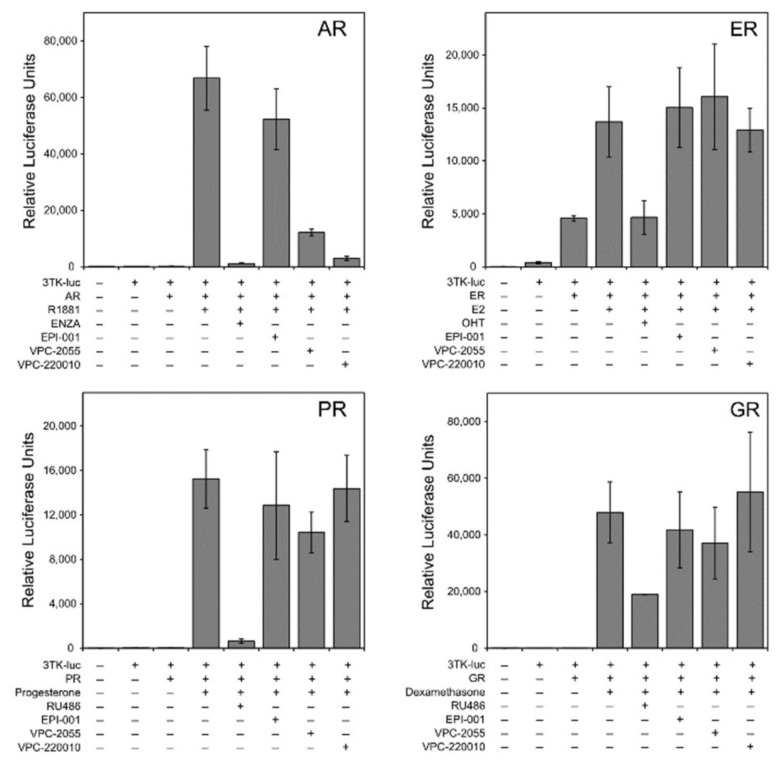
The selectivity inhibition profile of VPC-220010 against AR, ER, PR, and GR. Cells were transiently transfected with each nuclear receptor and suitable reporter plasmids before treatment with agonist and 20 uM of antagonists of EPI-001, VPC-2055, and VPC-220010.

Multiple sequence alignment of related nuclear receptors shows marked amino acid diversity in the NTDs of AR, estrogen (ER), glucocorticoid (GR) and progesterone (PR) receptors. To rule out cross-reactivity with other receptors, we performed a reporter transcription assay for each of these nuclear receptors. We used reporter constructs containing the appropriate response regions for each nuclear receptor: ARR3tk for AR/GR/PR and estrogen response element for ER. Each receptor was stimulated with their specific agonist; the synthetic androgen methyltrienolone (R1881) for AR, estradiol (E2) for ER, progesterone for PR, and dexamethasone for GR (Figure 4). As expected, we observed an increase in transcriptional reporter activity for each nuclear receptor when treated with its own specific agonist. This activity was inhibited when treated with known antagonists targeting the nuclear receptor including enzalutamide (MDV-3100) for AR, 4-hydroxytamoxifen (OHT) for ER, mifepristone (RU-486) for GR and PR. Similarly, VPC-220010 also inhibited AR transcriptional activity in this assay but had no significant effect on the transcription of ER, GR and PR (Figure 4). Overall, these results demonstrate that the VPC-220010 activity is specific to AR-NTD.

### 3.4. VPC-220010 Reduces DNA Binding and Expression of Androgen Response Genes

**Figure 5:**
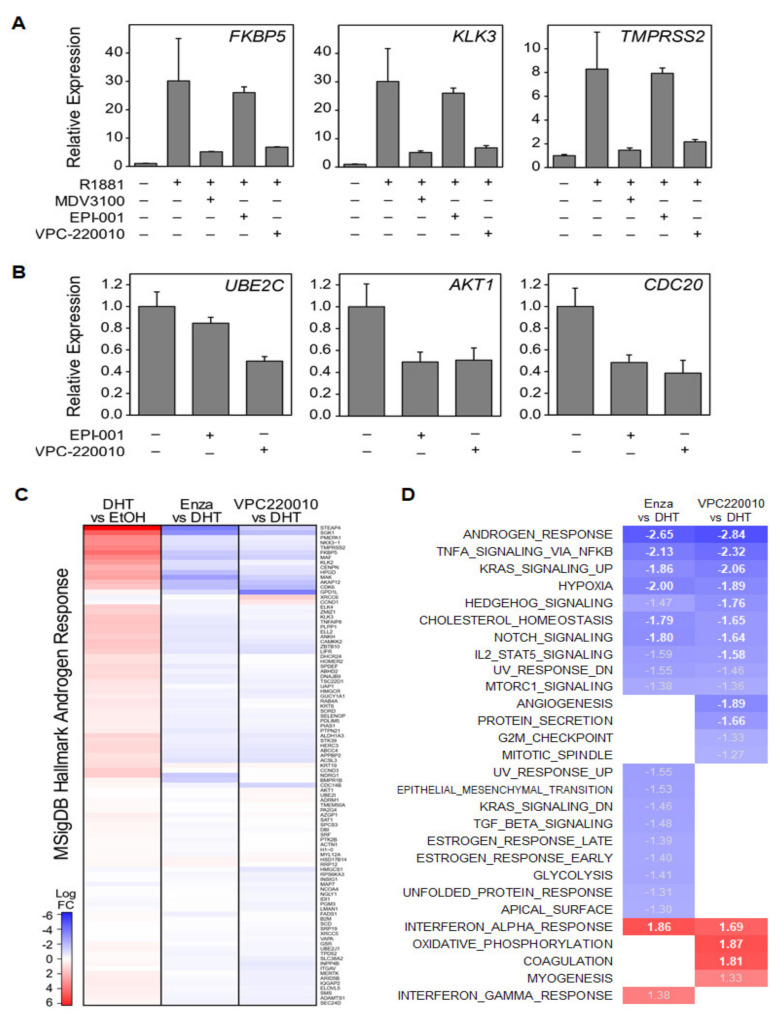
VPC-220010 inhibits the transcriptional activity of (**A**) canonical full-length AR-regulated genes KLK3, TMPRSS2 and FKBP5 in LNCaP cells; (**B**) ARV7-regulated genes UBE2C, AKT1 and CDC20 in ARV7 expressing cells 22rv1. (**C**) RNA-seq of VPC-220010 treated LNCaP cells demonstrated strong downregulation of the hallmark androgen response gene signature; (**D**) GSEA analysis of VPC-220010 treated LNCaP. A negative enrichment of genes associated with angiogenesis and protein secretion was observed in VPC-220010 but not ENZA.

Next, we characterized the impact of VPC-220010 on AR- and ARV7-mediated gene expression using qPCR. We determined that VPC-220010 significantly reduced canonical AR-regulated genes *KLK3*, *TMPRSS2* and *FKBP5* as efficiently as enzalutamide and markedly better than EPI-001 (Figure 5A). Furthermore, the ARV7-regulated genes *UBE2C, AKT1* and *CDC20* were inhibited by VPC-220010 and EPI-001 in ARV7 expressing cells 22Rv1 (Figure 5B). Next, we looked at the effect of VPC-220010 on the global transcriptome in LNCaP cells using RNA sequencing. Matching our qPCR results, *KLK3 TMPRSS2,* and *FKBP5* were all downregulated when treated with VPC-20010. In agreement with these findings, genes known to be androgen-regulated were strongly downregulated by VPC-220010 (Figure 5C). Gene set enrichment analysis showed significant negative enrichment in the Androgen Response gene set, with a greater normalized enrichment score than that of enzalutamide treatment (Figure 5D). Interestingly, treatment with VPC-220010 also resulted in negative enrichment of genes associated with angiogenesis and protein secretion (Figure 5D). Overall VPC-220010 robustly inhibited AR-mediated transcription in PCa models.

### 3.5. VPC-220010 Disrupts Interactions of AR with Its Cofactors

**Figure 6:**
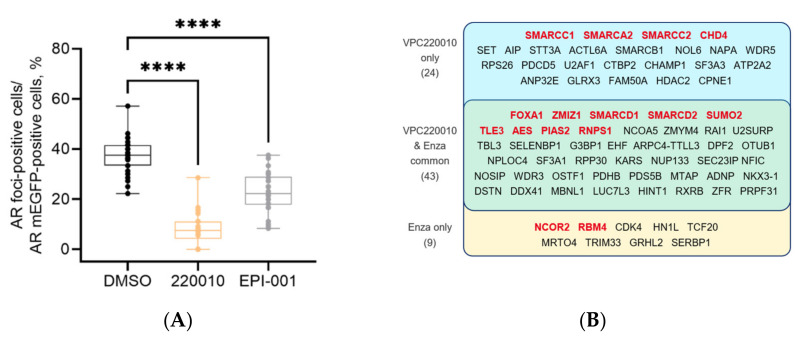
VPC-220010 impacts the formation of phase condensates and recruitment of co-regulatory protein (**A**) Effect of 10 µM VPC-220010 or EPI-001 on foci formation in AR-mEGFP transfected LNCaP cells. Condensates were quantified by using confocal microscopy. The dotted blot presents the data from at least 30 cells from three independent experiments. *p* values are indicated by stars: **** < 0.0001. (**B**) RIME results of LNCaP cells treated with EtOH, DHT, VPC-220010 or ENZA. The co-regulatory proteins inhibited by treatment of both VPC-220010 and ENZA or each individual compound are listed above. Genes highlighted in red have been previously identified as AR interacting proteins.

The AR-NTD is well known to recruit numerous co-activator proteins that are required for gene expression [24]. Recently, it has been reported that in various cancers transcription factors with intrinsically disordered (ID) activation domains can form phase-separated condensates with co-activators at super-enhancers to promote robust transcription of oncogenic programs [25,26,27]. We recently demonstrated that the AR forms these liquid-like foci in prostate cancer models upon androgen stimulation that correlate with its transcriptional activity [28]. Thus, we evaluated if VPC-220010 can disrupt the formation of phase-separated condensates as mean of transcriptional regulation. LNCaP cells transfected with AR-mEGFP (a non-dimerizing form of EGFP) were stimulated with DHT for 2 h to ensure AR nuclear translocation and foci formation, and then treated with 10 µM of the VPC-220010 or EPI-001 for an additional 2 h. VPC-220010 significantly reduced foci formation of AR in LNCaP cells, stronger than the known NTD inhibitor EPI-001 (Figure 6A).

As phase-condensates help to recruit additional proteins, we next questioned what coregulators would be impacted by VPC-220010 treatment. Therefore, we performed rapid immunoprecipitation mass spectrometry of endogenous protein (RIME), an approach that combines immunoprecipitation with mass-spectrometry to identify all AR interacting proteins on chromatin [29]. Using this on LNCaP cells treated with either DMSO + EtOH, DMSO + DHT, enzalutamide + DHT or VPC-220010 + DHT, we identified 111 AR-interacting proteins when cells were incubated with DHT, of which 20 were previously published interactors such as FOXA1 CHD4 and NCOR2 (Supplementary Table S2) [18]. When treated with VPC-220010, 67 of these interactions were lost, including 13 of the known AR interactors (Figure 6B). In comparison, enzalutamide disrupted 52 interactions, 11 of which were known AR interactors. The known AR interactions disrupted by VPC-220010 include CHD4, FOXA1, ZMIZ1, SMARCA2, SMARCC1, SMARCC2, SMARCD1, SMARCD2, SUMO2, TLE3, AES, PIAS2, and RNPS1 (Figure 6B). Several of these, such as the chromatin remodelers CHD4 and members of the SWIF/SNIF complex (SMARCA2, SMARCC1, SMARCC2), were uniquely disrupted by VPC-220010, and not by enzalutamide (Figure 6B). Overall, these results demonstrate that VPC-220010 disrupt phase condensate formation and impacts AR co-regulator interactions.

## 4. Discussion

The NTD is an attractive drug target for treating both early stage PCa and CRPC, as both ligand-dependent and -independent transcriptional activity of the AR is mediated, almost exclusively, by the N-terminal Tau1 and Tau5 regions. Targeting the AR NTD can therefore effectively bypass many mechanisms of resistance in CRPC including ARVs and point mutations. However, the identification of small molecules targeting the AR-NTD has proven to be an elusive goal as the NTD is a disordered protein domain.

Based on our early work, we found that the small molecule compound VPC-2055 was inhibiting the AR through interaction with the NTD. With the help of our in-house computer-aided drug discovery (CADD) program, we developed its derivative VPC-220010 based on this novel scaffold. In this work we demonstrate that VPC-220010 can effectively inhibit the transcriptional activity of both full-length AR, and LBD-truncated variant ARV7 (Figure 2A,B). VPC-220010 reduces the viability of AR-dependent prostate cancer cell lines LNCaP and 22Rv1, while having no significant effect on an AR negative cell line (Figure 2C,D). Importantly, this compound is selective for AR-NTD and does not significantly affect the closely related steroid receptors ER, PR, and GR (Figure 3).

The AR-NTD mediates transcriptional activity through interactions with co-regulatory proteins [7,8,30]. These interactions are proposed to occur via the formational of biological phase condensates at critical *cis*-regulatory enhancer elements [25]. When cells were treated with VPC-220010 we observed a significant decrease in the number of phase condensates suggesting that the AR-NTD plays a critical role in either the formation or stabilization of these transcriptional hubs. Using RIME, we demonstrated that VPC-220010 treatment dramatically altered the AR interactome including the loss of critical co-regulators such as FOXA1 and ZMIZ1 [31] (Figure 6). In support of the novel mechanism of NTD antagonists, VPC-220010 disrupted several protein interactions that were not altered by enzalutamide treatment; such as CHD4, a central subunit of the nucleosome remodeling and histone deacetylation (NuRD) complex, and several SWI/SNF complex proteins such as SMARCC1, SMRCC2, and SMARCA2 (Figure 6). These SWIF/SNF components are commonly upregulated in prostate cancer, and correlate with tumor recurrence and dedifferentiation [17] by localizing at genomic regions marked with the active chromatin histone mark H3K27Ac [32]. SMARCA2 specifically, is known to be critical to AR dependent transcriptonal activity [33].

In addition to improved potency, VPC-220010 also has improved metabolic stability compared to VPC-2055 (2×). However, further medicinal chemistry optimization is needed to make this scaffold stable enough to proceed to potential preclinical studies. Importantly, VPC-220057 is a 100-fold more stable in human microsomes than VPC-220010, albeit 10-fold less potent (Table 1). Study of VPC-220057 scaffold could lead to a VPC-220010- and VPC-220057-derivative that combines their best potency and stability. The corresponding efforts are under way.

## 5. Conclusions

This study broadens the repertoire of compounds targeting the AR NTD and provides useful insight for further development of novel antiandrogens with unique and novel mechanisms of action. The AR-NTD small molecule inhibitor prototype identified in this work possesses promising clinical utility for treating resistant forms of late-stage prostate cancer. Such drugs would be of immense value for CRPC patients that do not respond to conventional AR inhibitors.

## Figures and Tables

**Table 1:**
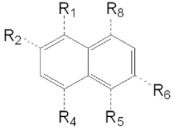
Efficacy and microsomal stability of the active AR-NTD inhibitors.

VPCID	R1	R2	R4	R5	R6	R8	eGFP IC_50_ (μM)	PSA IC_50_ (μM)	PC3-iV7 IC_50_ (μM)	T_1/2_ (min.)
220009	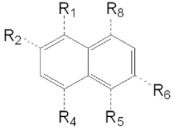			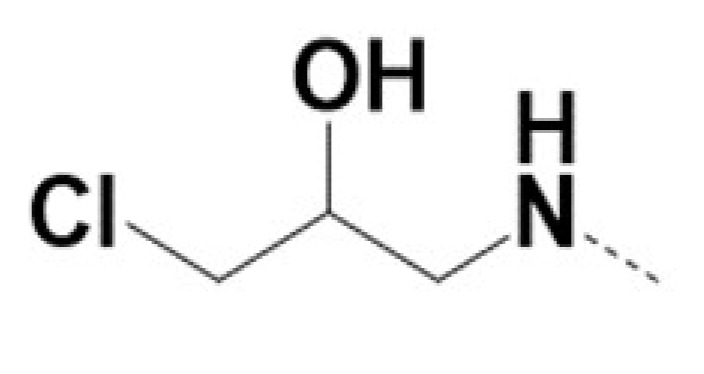			1	1	NA	
220010	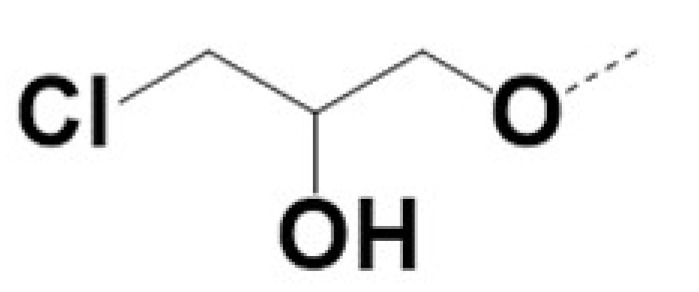			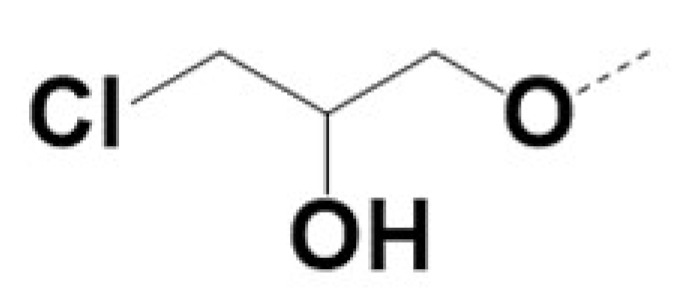			0.5	0.7	2.3	30
220027	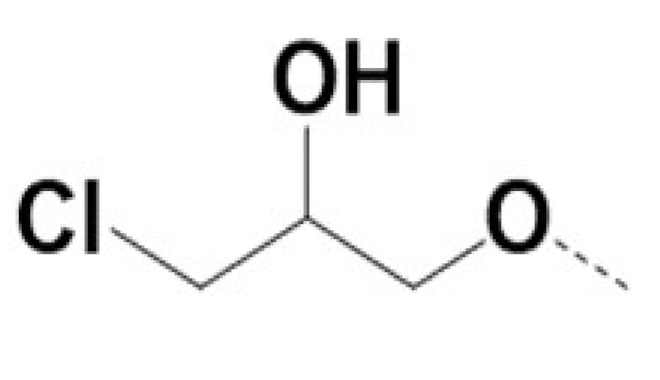	Br		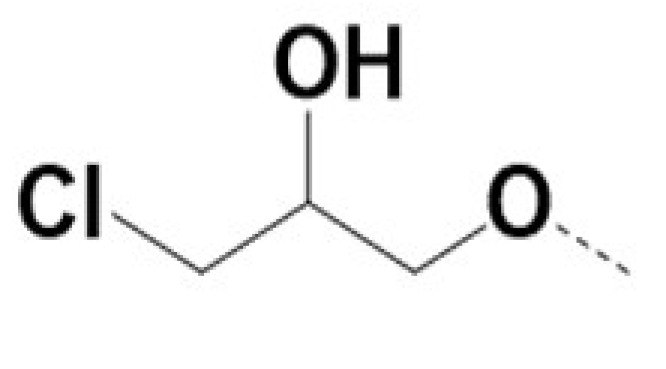	Br		3	4	4	TBD
220028	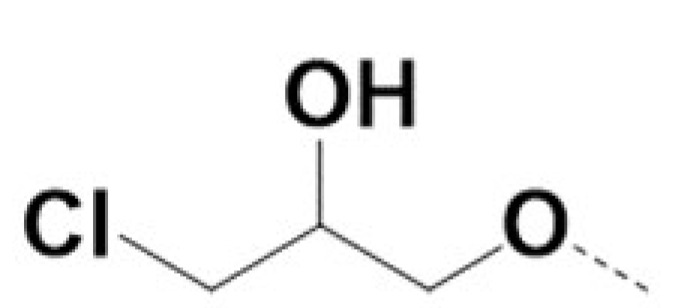		Br	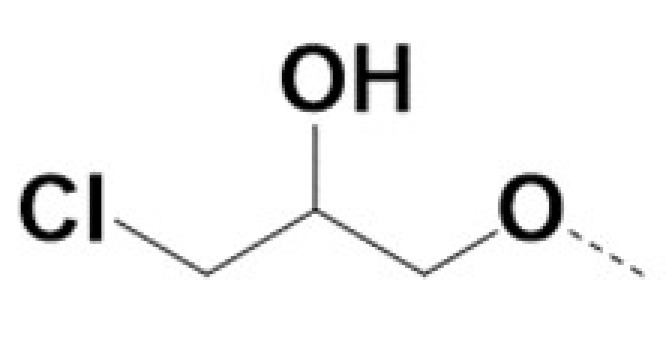		Br	0.4	0.4	3	NA
220054	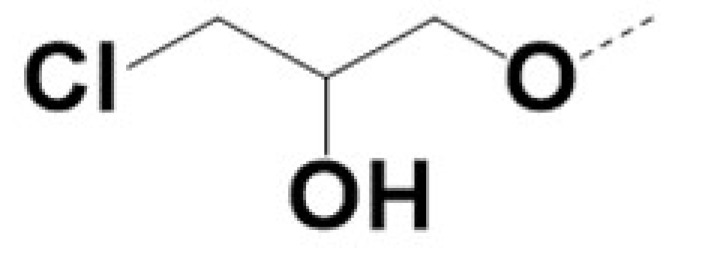	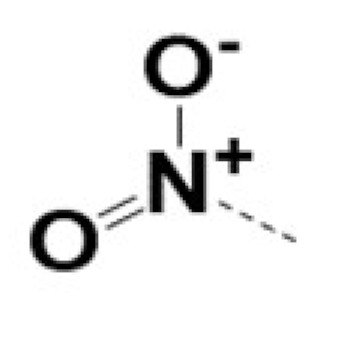		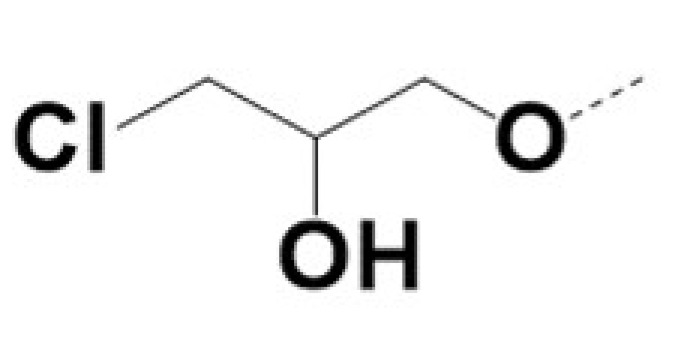			5	5	3	NA
220057	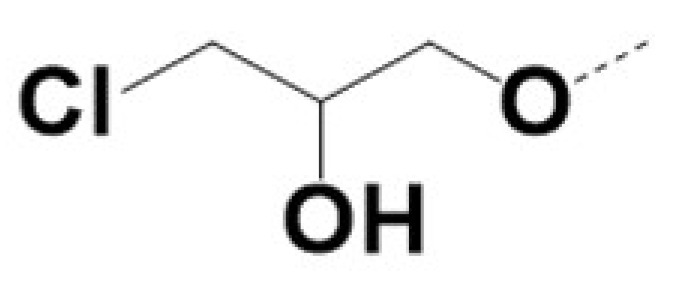		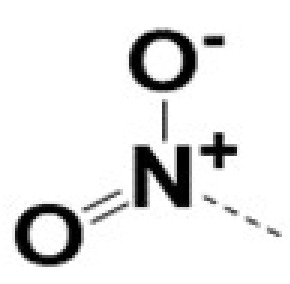	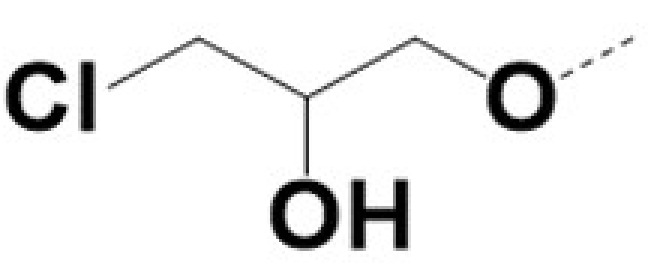		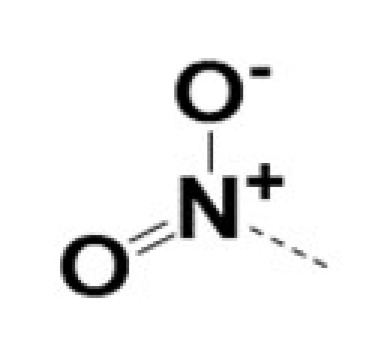	10	8	8	462
220062	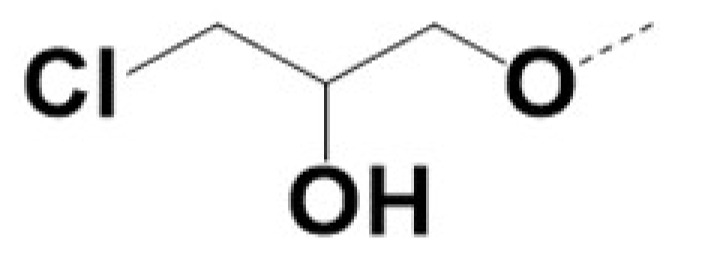		Br	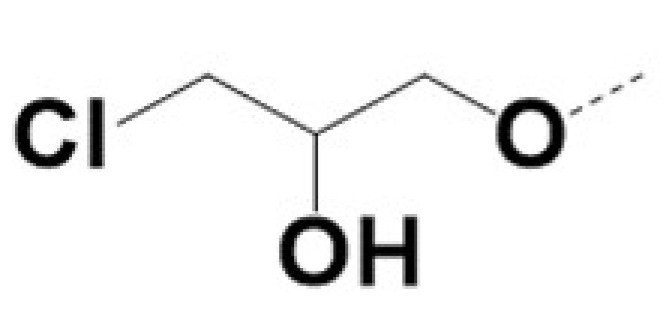			0.5	0.5	3	36
220075		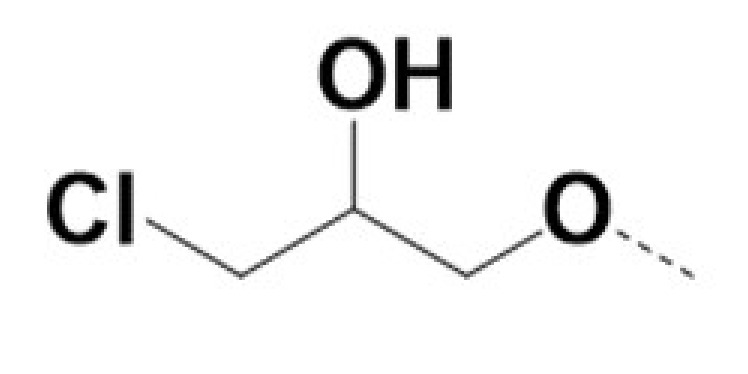			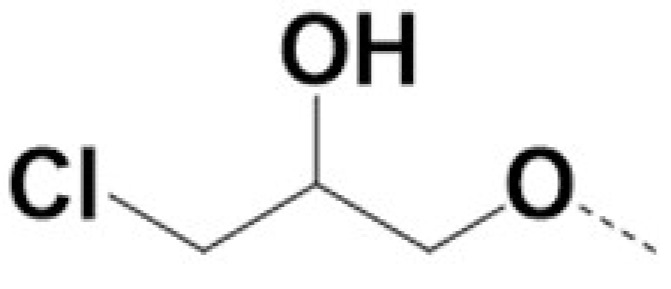		0.5	0.5	12	18
RR-220010	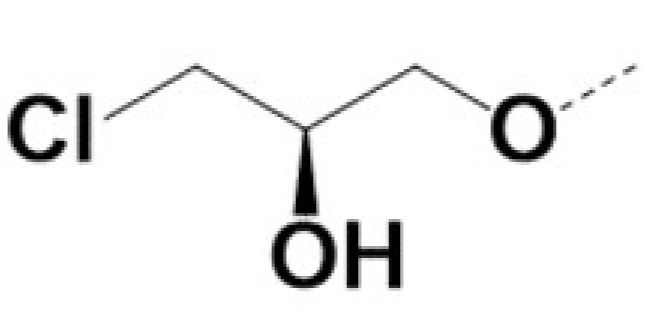			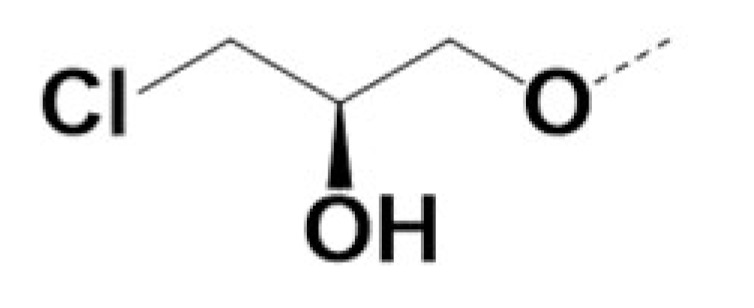			0.7	0.7	5	11
SS-220010	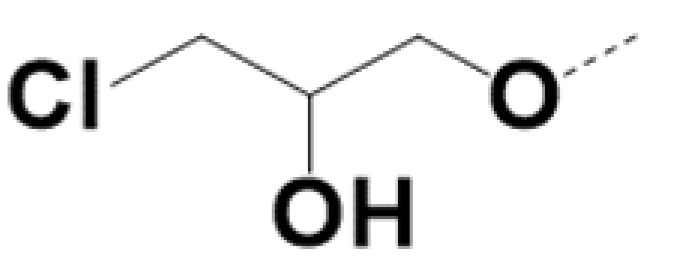			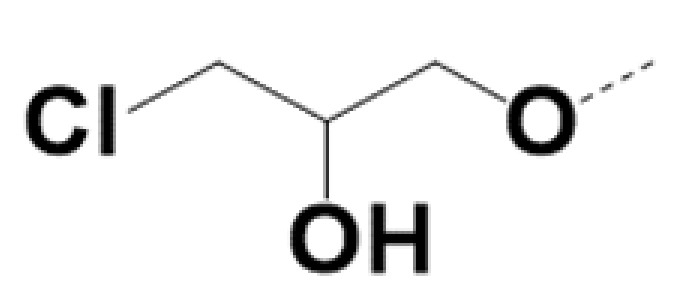			0.7	0.6	6	12
RS-220010	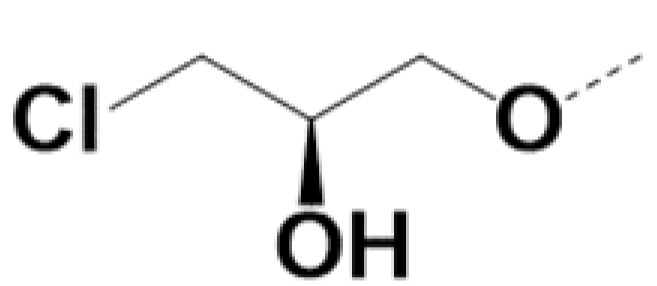			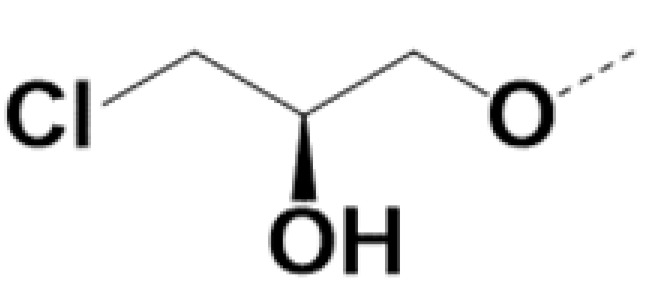			0.85	0.79	5	15

## Data Availability

Not applicable.

## Supplementary Materials

The following are available online at https://www.mdpi.com/article/10.3390/cancers13143488/s1, Table S1. Result Files, Table S2: List of AR-interacting proteins identified by RIME.
