# Supplementary material for: Development of an Androgen Receptor Inhibitor Targeting the N-Terminal Domain of Androgen Receptor for Treatment of Castration Resistant Prostate Cancer
Source: Cancers (Basel). 2021 Jul 12;13(14):3488. doi: 10.3390/cancers13143488 (PMC8304761; doi:10.3390/cancers13143488)
Supplement: Supplementary file 1 [file cancers-13-03488-s001.zip › Supplementary materials.pdf]

Supplementary Materials:

1) List of Primers:

FKPB5: F 5′-CGCAGGATATACGCCAACAT -3'

R 5′- GAAGTCTTCTTGCCCATTGC -3'

KLK3: F 5′-CCAAGTTCATGCTGTGTGCT -3'

R 5′- CCCATGACGTGATACCTTGA -3'

TMPRSS2: F 5′-TACTCTGGAAGTTCATGGGC -3'

R 5′- GTCATCCACTATTCCTTGGCT -3'

FKBP52: F 5′-GAATACAGACTCGCGGTGAA -3'

R 5′- CAGTGCAACCTCCACGATAG -3'

UBE2C: F 5'- ACC TTT TCA AAT GGG TAG GG -3'

R 5'- GAC ATC ATA CAG GGC AGA CC -3'

Cdc20: F 5'-GCA CAG TTC GCG TTC GAG A -3'

R 5'-CTG GAT TTG CCA GGA GTT CGG -3'

AKT1: F 5'- AGC GAC GTG GCT ATT GTG AAG -3'

R 5'- GCC ATC ATT CTT GAG GAG GAA GT -3'

2) The synthesis of racemic and enantiomeric VPC-220010

Reagents and solvents were purchased from commercial suppliers (Sigma-Aldrich, Apollo Scientific, Fisher, Acros Organic, and Alfa Aesar) and used without further purification. Chromatography solvents were HPLC grade and also used without further purification. Column chromatography was performed on Combiflash RF 200 using Kieselgel Merck 60 (230–400 mesh) as the stationary phase. NMR spectra were recorded on a Varian Gemini 2000 using an internal deuterium lock. LC-MS and GC-MS spectra were recorded on an Agilent 1100 LCMSD SL instrument (electrospray ionization (ESI)) or Agilent GC7820A/MSD5977B system (electron impact ionization (EI), ionization energy – 70 eV). Internal normalization method allowing the quantitative determination of a sample component by relating its peak area to that of the total peak was used. Conversion measurements were performed using the same GC-MS instrument (column: HP-5ms UI, 30 m - 0.25 mm, 0.25 μm; carrier gas: helium at 1 mL/min; temperatures: injector – 250 °C, oven program – 50 °C initial temperature for 1 min, then ramp to 300 °C at 20 °C/min, then hold final temperature for 5 min; MSD transfer line – 280°C, MSD source – 230°C, MSD quad – 150°C; injection parameters: split ratio 200:1, 0.5 μL injected; MS parameters: mass scan range – 35–550, ionization energy – 70eV).

VPC-220010 has two chiral centers that can generate three enantiomers of RR-220010, RS-220010, and SS-220010 shown in Figure 1.

The synthesis of the racemic VPC-220010 is shown in Scheme 1. The dioxane solution of HCl (4M, 1 mL) was added to a solution of the corresponding epoxide (0.1 g, 0.2-0.4 mmol) in dioxane (1 mL). The reaction mixture was stirred at room temperature for 30 min. Then the solvent was removed under reduced pressure. The crude product was purified by flash chromatography on silica gel.

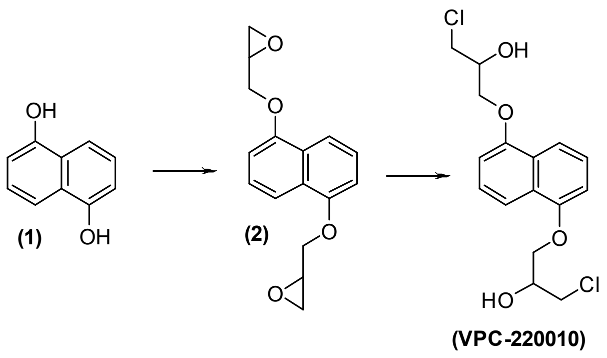

Scheme 1. Synthesis of the racemic VPC-220010

VPC-220010: Yield 120 mg (95%). ^{1}H NMR (400 MHz, CD_3OD) δ 7.86 (d, *J* = 8.3 Hz, 2H), 7.37 (t, *J* = 8.0 Hz, 2H), 6.95 (d, *J* = 7.7 Hz, 2H), 4.28 (dd, *J* = 10.2, 5.0 Hz, 2H), 4.25 – 4.19 (m, 4H), 3.84 (ddd, *J* = 34.0, 11.3, 5.1 Hz, 4H). MS (ESI) 345.0, 347.0 (M+H^{+}).

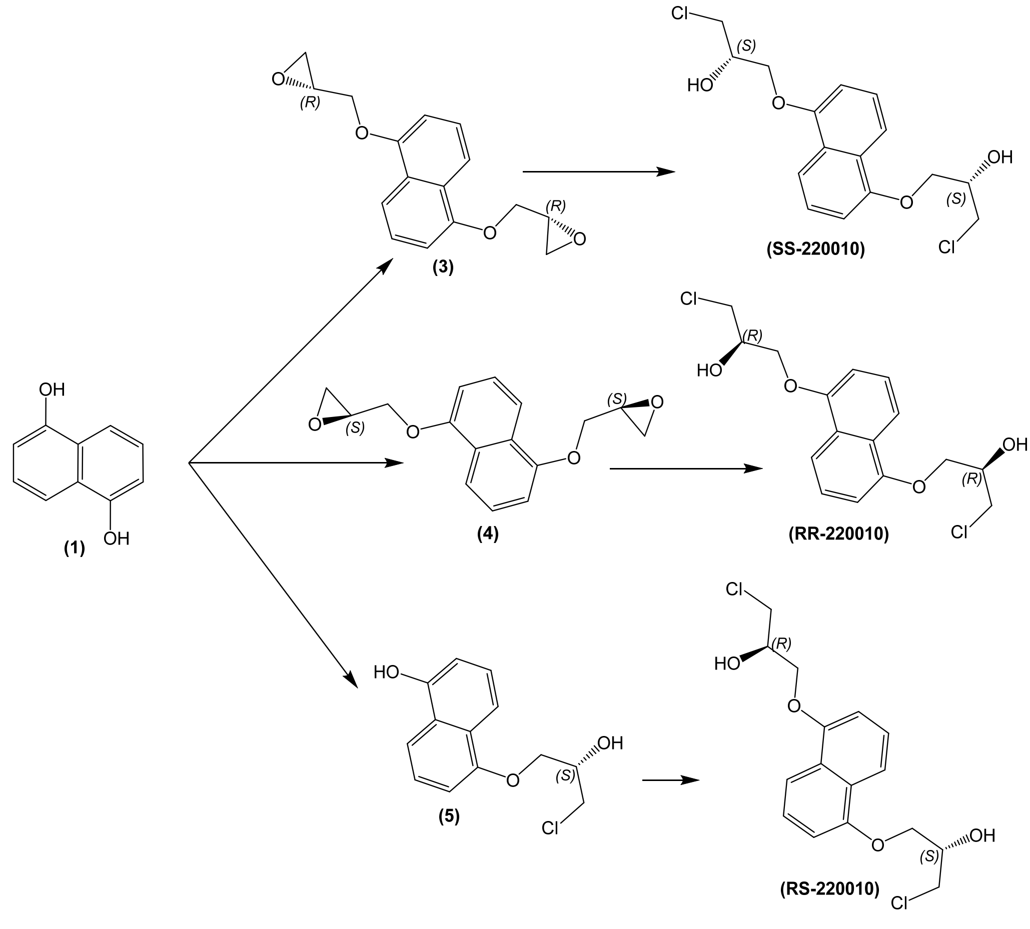

Scheme 2. Chiral syntheses of RR-220010, RS-220010, and SS-220010

The syntheses of RR-220010, RS-220010, and SS-220010 were shown in Scheme 2. General procedure for preparation of enantiomeric compounds (**3**) and (**4**). Potassium carbonate (3.5 g, 25 mmol) was added to a solution of naphthalene-1,5-diol (1 g, 6.25 mmol) in dry acetone (20 mL) under argon atmosphere. Then the corresponding 2-(chloromethyl)oxirane enantiomer (1.4 g, 18.75 mmol) was added in one portion. The reaction mixture was heated to reflux overnight. Upon reaching room temperature the mixture was subjected to filtration. The filtrate was washed with dry acetone (3×10 mL) and the collected liquid part was evaporated under reduced pressure. The crude product was purified by flash chromatography on silica gel.

Compounds (**3**) and (**4**), Yield 120 mg (7%) ^{1}H NMR (400 MHz, CD_3CN) δ 7.86 (d, *J* = 8.6 Hz, 2H), 7.40 (t, *J* = 8.0 Hz, 2H), 6.97 (d, *J* = 10.6 Hz, 2H), 4.33 – 4.24 (m, 2H), 4.21 (d, *J* = 5.2 Hz, 4H), 3.84 (ddd, *J* = 17.0, 11.3, 5.2 Hz, 4H). MS (EI) 272.1 (M^+).

General procedure for preparation of compounds (SS-220010), (RR-220010), and (RS-220010). The dioxane solution of HCl (4M, 1 mL) was added to a solution of the corresponding epoxide (0.1 g, 0.36 mmol) in dioxane (1 mL). The reaction mixture was stirred at room temperature for 30 min. Then the solvent was removed under reduced pressure. The crude product was purified by flash chromatography on silica gel.

Yields (SS-220010) 40 mg (32%), (SS-220010) 35 mg (28%), (RS-220010) 35 mg (28%). ^{1}H NMR (400 MHz, CD_3OD) δ 7.86 (d, *J* = 8.3 Hz, 2H), 7.37 (t, *J* = 8.0 Hz, 2H), 6.95 (d, *J* = 7.7 Hz, 2H), 4.28 (dd, *J* = 10.2, 5.0 Hz, 2H), 4.25 – 4.19 (m, 4H), 3.84 (ddd, *J* = 34.0, 11.3, 5.1 Hz, 4H). MS (ESI) 345.0, 347.0 ($M+H^+$).

Compound (**5**). NaOH (0.3 g, 7.5 mmol) was added to a suspension of naphthalene-1,5-diol (1 g, 6.25 mmol) in deionized water (20 mL). The mixture was stirred at room temperature for 20 min. Then S-2-(chloromethyl)oxirane (0.66 g, 7 mmol) was added in one portion and the reaction mixture was stirred overnight. The excess of NaOH was neutralized with an aqueous solution of citric acid (0.1 M) to pH = 4. The resulting mixture was extracted with ethyl acetate (3×20 мл). The organic layers were combined, dried over Na_2SO_4, and the solvent was removed under reduced pressure. The resulting oily residue was purified by flash chromatography on silica gel.

Compound (**5**) Yield 160 mg (10%) ^{1}H NMR (400 MHz, CD_3OD) δ 7.77 (d, *J* = 8.5 Hz, 1H), 7.73 (d, *J* = 8.5 Hz, 1H), 7.34 – 7.28 (m, 1H), 7.28 – 7.21 (m, 1H), 6.90 (d, *J* = 7.7 Hz, 1H), 6.83 (d, *J* = 7.5 Hz, 1H), 4.29 (dt, *J* = 10.3, 5.1 Hz, 1H), 4.23 (t, *J* = 5.0 Hz, 2H), 3.84 (ddd, *J* = 35.5, 11.3, 5.9 Hz, 3H). MS (ESI) 251.2 ($M−H^+$).

Compound (**RS-220010**). NaOH (35 mg, 0.87 mmol) was added to a suspension of compound (**5**) (150 mg, 0.7 mmol) in deionized water (3 mL). The mixture was stirred at room temperature for 20 min. Then *R*-2-(chloromethyl)oxirane (78 mg, 0.85 mmol) was added in one portion and the reaction mixture was stirred overnight. Then the reaction mixture was taken up with ethyl acetate (3×5 mL). The organic layers were combined, dried over Na_2SO_4, and the solvent was removed under reduced pressure. The resulting oily residue was purified by flash chromatography on silica gel.

Yield 115 mg (60%) ^{1}H NMR (400 MHz, CD_3CN) δ 7.86 (d, *J* = 8.6 Hz, 2H), 7.40 (t, *J* = 8.0 Hz, 2H), 6.97 (d, *J* = 10.6 Hz, 2H), 4.33 – 4.24 (m, 2H), 4.21 (d, *J* = 5.2 Hz, 4H), 3.84 (ddd, *J* = 17.0, 11.3, 5.2 Hz, 4H). MS (EI) 272.1 (M^+).

Supplementary Table 1: DESeq2 Result Files

Supplementary Table 2: List of AR-interacting proteins identified by RIME
